# A computational approach based on the colored Petri net formalism for studying multiple sclerosis

**DOI:** 10.1186/s12859-019-3196-4

**Published:** 2019-12-10

**Authors:** Simone Pernice, Marzio Pennisi, Greta Romano, Alessandro Maglione, Santina Cutrupi, Francesco Pappalardo, Gianfranco Balbo, Marco Beccuti, Francesca Cordero, Raffaele A. Calogero

**Affiliations:** 1 Department of Computer Science, University of Turin, Turin, Italy; 2 Department of Mathematics and Computer Science, University of Catania, Catania, Italy; 3 Department of Clinical and Biological Sciences, University of Turin, Orbassano, Italy; 4 Department of Drug Sciences, University of Catania, Catania, Italy; 5 Department of Molecular Biotechnology and Health Sciences, University of Turin, Turin, Italy

**Keywords:** Multiple sclerosis, Computational models, Colored petri nets, Sensitivity analysis

## Abstract

**Background:**

Multiple Sclerosis (MS) is an immune-mediated inflammatory disease of the Central Nervous System (CNS) which damages the myelin sheath enveloping nerve cells thus causing severe physical disability in patients. Relapsing Remitting Multiple Sclerosis (RRMS) is one of the most common form of MS in adults and is characterized by a series of neurologic symptoms, followed by periods of remission. Recently, many treatments were proposed and studied to contrast the RRMS progression. Among these drugs, daclizumab (commercial name Zinbryta), an antibody tailored against the Interleukin-2 receptor of T cells, exhibited promising results, but its efficacy was accompanied by an increased frequency of serious adverse events. Manifested side effects consisted of infections, encephalitis, and liver damages. Therefore daclizumab has been withdrawn from the market worldwide. Another interesting case of RRMS regards its progression in pregnant women where a smaller incidence of relapses until the delivery has been observed.

**Results:**

In this paper we propose a new methodology for studying RRMS, which we implemented in GreatSPN, a state-of-the-art open-source suite for modelling and analyzing complex systems through the Petri Net (PN) formalism. This methodology exploits: (a) an extended Colored PN formalism to provide a compact graphical description of the system and to automatically derive a set of ODEs encoding the system dynamics and (b) the Latin Hypercube Sampling with PRCC index to calibrate ODE parameters for reproducing the real behaviours in healthy and MS subjects.To show the effectiveness of such methodology a model of RRMS has been constructed and studied. Two different scenarios of RRMS were thus considered. In the former scenario the effect of the daclizumab administration is investigated, while in the latter one RRMS was studied in pregnant women.

**Conclusions:**

We propose a new computational methodology to study RRMS disease. Moreover, we show that model generated and calibrated according to this methodology is able to reproduce the expected behaviours.

## Background

Multiple Sclerosis (MS) is a chronic and potentially highly disabling disease with considerable social impacts and economic consequences. In Europe it is the leading cause of non-traumatic disabilities in young adults, since more than 700,000 EU people suffer from MS [1].

Multiple sclerosis is an inflammatory autoimmune disease in which the patient’s immune system reacts against itself by damaging CNS nerve cells - i.e. compromising the ability of the neurons to send electrical signals - resulting in a progression of physical handicap until complete paralysis within 25 years in more than 30% of patients [2].

In literature four courses of MS are identified: Relapsing-Remitting MS (RRMS), Secondary Progressive MS (SPMS), Primary Progressive MS (PPMS), and Progressive Relapsing MS (PRMS). Among them the RRMS is the most common course since it is diagnosed in about 85% of MS cases. It is characterized by episodes of neurological dysfunction (i.e. relapses) followed by a complete or partial recovery (i.e. remissions). Unfortunately, within 25 years RRMS usually changes to SPMS (in about 90% of cases) increasing the severity of the disease. [1]

Despite the etiology of MS is unknown, researchers agree that also environmental factors can act as triggers of MS, leading to the inflammatory process in the Central Nervous System (CNS). In particular, viruses may play a role in MS pathogenesis acting as such environmental triggers. Some studies linked MS with Epstein Barr Virus (EBV) infection due to the presence of higher titers of EBV antibodies in MS patients compared to age-matched controls [3].

Besides environmental factors, physiological factors also impact on the outcome of the MS disease. In particular, pregnancy represents a period of immune tolerance for patients that has important consequences on the relapse rate [4]. Indeed, pregnancy condition seems to have beneficial effects on women patients which have been associated with fewer relapses in RRMS. This phenomenon has been related with an increase in a particular type of immune cells, the Regulatory T lymphocytes cells (Treg), which confers fetal tolerance and thus shows a protective effect of pregnancy to patients [5].

In the last two decades the advances in the understanding of the immune pathogenesis of MS and the advent of Monoclonal Antibodies (mAb) allowed researchers to define novel treatments against this disease. In particular mAb are powerful new tools to modify the course of MS based on a molecular targeted approach. Indeed, they are potentially able to break the immune cascade of events that brings to the autoimmune reaction causing the myelin loss. Therefore, these treatments that include several mAbs such as Natalizumab, Rituximab, and Alemtuzumab, constitute nowadays the most effective first and second line treatments in the therapy of MS [6–8].

Moreover, when the first and second line treatments provide an inadequate response in patients, daclizumab (DAC) treatment [9] represented the only third line treatment to be used as a valid alternative. Differently from the other mAbs, DAC is a humanized monoclonal IgG1 antibody tailored against InterLeukin-2 Receptor (IL2R), thus able to break the autoimmune reaction by suppressing the immune cells expansion.

The basic mechanism of MS is, however, not fully understood yet and, despite its promising results, in 2018 DAC was withdrawn from the EU marketing authorization process due to the observation of twelve cases of patients who developed, after the beginning of the treatment, serious immune-mediated adverse reactions at the level of the CNS, including encephalitis and meningoencephalitis. More studies are needed to understand these effects as well as to explain why women affected by MS seem to improve when they become pregnant and during the pregnancy period. To improve our understanding of these phenomena, in this paper we extend the RRMS models presented in [10–12] proposing a new computational methodology to analyse the RRMS behaviour. Hence, we firstly describe how the Extended Stochastic Symmetric Net (ESSN) formalism can be efficiently used to derive a graphical and parametric description of the system under study. Then, we show how the system of Ordinary Differential Equations (ODEs), that can be automatically derived from an ESSN model, reproduces the disease dynamics and how uncertainty and sensitivity analysis can be used to make more robust the results provided by the model. Finally we tested the proposed methodology constructing a model which allows to represent two different scenarios where the effect of the daclizumab administration is investigated and the RRMS in pregnant women is considered.

## Methods

### Introduction to petri net formalism

In this section we provide an intuitive introduction to the formalism used to model and analyze our case study. The Petri Net formalism is firstly introduced, then a specific type of high-level extension, called Stochastic Symmetric Net (SSN) [13], is described. After, the technique used to derive the qualitative properties of systems modelled with this formalism is discussed showing how these results can be computed efficiently using a fluid approximation. Then a new extension of the SSN formalism, called ESSN, is introduced to deal easily with more complex biological laws different by Mass Action one. Finally, in the last part of this section we describe how the model sensitivity analysis can be carried out using a sampling-based method.

### Petri net and stochastic symmetric net

Petri Net (PN) [14] and their extensions are well-known computational and mathematical formalisms which provide a graphical intuitive and formal description of the important features of the system under study. They allow the use of different analysis techniques to derive the qualitative and quantitative properties of a system.

**Fig. 1:**
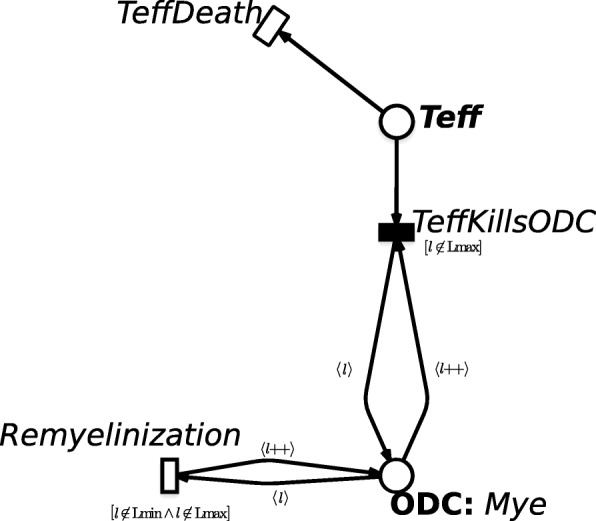
Example of SSN. Example of SSN representing the Effector T cells (place on the top named as Teff) which damage the Oligodentrocytes cells (place on the bottom named as ODC), and their partially recovery of the lost myelin when the damage is not excessive. This is a sub net of the SSN represented in Fig. 2

PNs are bipartite directed graphs with two types of nodes, namely places and transitions. Places, correspond to state variables of the system and are graphically represented as circles. For instance, in Fig. 1 an example PN model is presented, where it is described i) the Effector T cells (Teff) attack to the myelin sheaths due to the structure similarity of the viral protein with myelin proteins, and ii) the Oligodentrocytes cells (ODC) recovery of the lost myelin when the damage is not irreversible. Indeed, these events play a central role in RRMS progression, and more details will be given in Sec. *Model description*. Here the *Teff* and *ODC* nodes are model places representing the Effector T cells and the Oligodentrocytes cells, respectively.

**Fig. 2:**
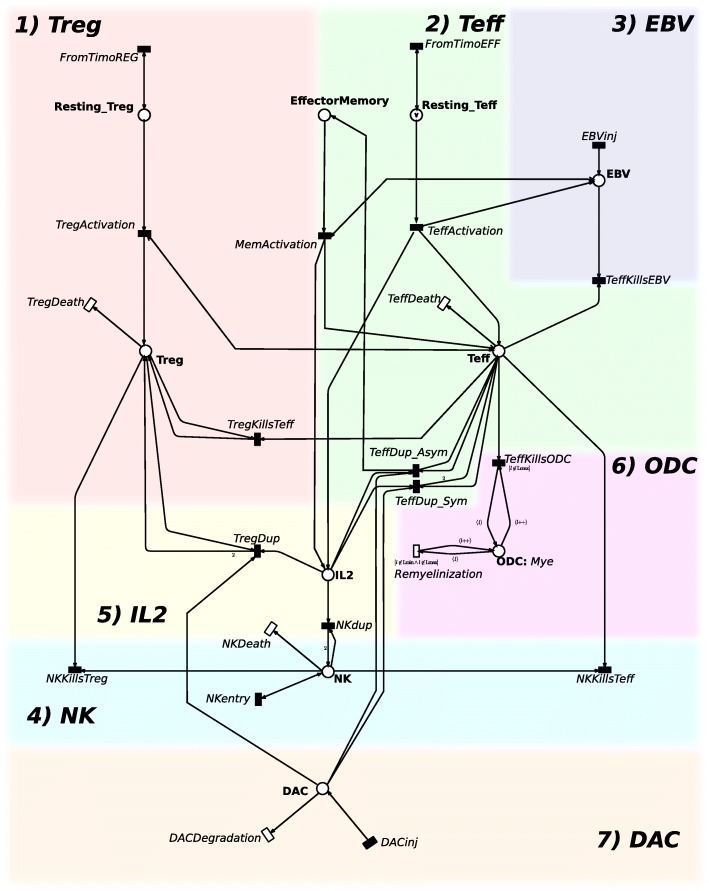
The RRMS model. The RRMS PN model is composed by places (graphically represented by circles) corresponding to cells or molecules, and by transitions (graphically represented by rectangles) corresponding to the interactions among the entities, injections or death of molecules. The RRMS model is composed by seven modules: Treg, Teff, EBV, NK, IL2, ODC and DAC

Differently, transitions correspond to the events that can induce a state change and are graphically represented as boxes. Referring again to Fig. 1, transitions are *TeffDeath*, *Remyelinization* and *TeffKillsODC* which simulate the Teff death, the ODC recovery, and the damages of the Teff over the ODC cells, respectively.

The arcs connecting places to transitions (and vice-versa) express the relation between states and event occurrences. Places can contain tokens, drawn as black dots. The state of a PN, called *marking*, is defined by the number of tokens in each place of the model.

The system evolution derives from the firing of enabled transitions, where a transition is enabled if and only if each input place contains a number of tokens greater or equal than a given threshold defined by the cardinality (multiplicity) of the corresponding input arc. Thus, the firing of an enabled transition removes a fixed number of tokens from its input places and adds a fixed number of tokens into its output places, according to the cardinality of its input/output arcs.

Among the PN generalisations proposed in literature, SSNs [13] extend PNs providing a more compact and readable representation of the system, thanks to the possibility of using tokens belonging to different classes and thus graphically represented in the models as dots of different colors.

In SSNs each place *p* has an associated color domain (a data type) denoted *cd*(*p*) and each token in a given place has an associated value defined by *cd*(*p*). Color domains are defined by the Cartesian product of elementary types called *color classes*$\mathcal {C}=\{C_{1},\ldots,C_{n}\}$, so that $\mathit {cd}(p)=C^{e_{1}}_{1} \times C^{e_{2}}_{2}\times \ldots \times C^{e_{n}}_{n}$ where *e*_*i*_ is the number of times *C*_*i*_ appears in *cd*(*p*). Color classes are finite and disjoint sets. They can be ordered (in this case a successor function is defined on the class, inducing a circular order among the elements in the class), and can be partitioned into (static) subclasses (e.g *C*_*i*,*j*_ is the i^*th*^ static subclass of the j^*th*^ color class).

In the example model represented in Fig. 1 the ODC color domain is defined by one color class, the myelination levels of ODC cells, named *Mye*. This is divided into 5 static subclasses (i.e. *L*_*min*_, *L*1, *L*2, *L*3 and *L*_*max*_) so that myelination level ranges from an irreversible damage (*L*_*min*_, no myelination) to no damages (*L*_*max*_, full myelination). Similarly, a color domain is associated with transitions and is defined as a set of typed variables, where the variables are those appearing in the functions labeling the transition arcs and their types are the color classes. For instance, the color domain of transition *Remyelinization*, representing the recovery of a ODC cell, is *Mye* and the variable characterizing its input arc is *l*∈*Mye*.

An instance of a given transition *t* is an assignment of the transition variables to a specific color of proper type. Hence, we use the notation 〈*t*,*c*〉 to denote an instance, where *c* is the assignment, also called binding. Moreover, a guard can be used to define restrictions on the allowed instances of a transition. A guard is a logical expression defined on the color domain of the transition, and its terms, called basic predicates, allow (*i*) to compare colors assigned to variables of the same type (*x*=*y*, *x*≠*y*); (*ii*) to test whether a color element belongs to a given static subclass (*x*∈*C*_*i*,*j*_); (*iii*) to compare the static sub-classes of the colors assigned to two variables (*d*(*x*)=*d*(*y*),*d*(*x*)≠*d*(*y*)).

The *marking* of an SSN is defined by the number of colored tokens in each place. For instance, a possible marking of the place *DAC* in Fig. 1, is 500 〈*Lmax*〉 corresponding to 500 ODC cells with a full myelination.

Each arc connecting a place *p* to a transition *t*, namely an input arc of *t*, is labeled with an expression defined by the function *I*[*p*,*t*]:*cd*(*t*)→*Bag*[*cd*(*p*)], where *Bag*[*A*] is the set of multisets built on set *A*, and if *b*∈*Bag*[*A*]∧*a*∈*A*, *b*[*a*] denotes the multiplicity of *a* in the multiset *b*. Similarly, each arc connecting a transition *t* to a place *p*, namely an output arc of *t*, is denoted by the function *O*[*p*,*t*]:*cd*(*t*)→*Bag*[*cd*(*p*)]. Thus, the evaluation of *I*[*p*,*t*] (resp. *O*[*p*,*t*]), given a legal binding of *t*, provides the multiset of colored tokens that will be withdrawn from (input arc) or will be added to (output arc) the place connected to that arc by the firing of such transition instance. Moreover, we denote with ^∙^**t** the set of input places of the transition *t* and with **t**^∙^ the set of output places of *t*, i.e. ^∙^**t**:={*p*∈*P*| ∃ *c*∈*cd*(*p*) *s*.*t*. *I*[*p*,**t**](*c*^′^)[*c*]>0} and **t**^∙^:={*p*∈*P*| ∃ *c*∈*cd*(*p*) *s*.*t*. *O*[*p*,**t**](*c*^′^)[*c*]>0}. In details, a transition instance 〈*t*,*c*〉 is enabled and can fire in an marking *m*, iff: (1) its guard evaluated on *c* is true; (2) for each place *p* we have that *I*[*p*,*t*](*c*)≤*m*(*p*), where ≤ is the comparison operator among multisets. We use the notation *E*(*t*,*m*) to denote the set of all instances of *t* enabled in marking *m*. The firing of the enabled transition instance 〈*t*,*c*〉 in *m* produces a new marking *m*^′^ such that, for each place *p*, we have *m*^′^(*p*)=*m*(*p*)+*O*[*p*,*t*](*c*)−*I*[*p*,*t*](*c*).

In the SSNs, the firing time of an enabled transition instance 〈*t*,*c*〉 is sampled from a negative exponential distribution whose rate is given by the function *ω*, i.e.

$$ \omega(t,c)= \left\{ \begin{array}{ll} r_{i} & \textit{cond}_{i}(c)\ i=1,\dots,n,\\ r_{n+1} & \textit{otherwise},\\ \end{array} \right.   $$

where *cond*_*i*_ are boolean and mutually exclusive expressions comprising standard predicates on the transition color instance. In this manner, the firing rate *r*_*i*_ of a transition instance can depend only on the static sub-classes of the objects assigned to the transition parameters and on the comparison of variables of the same type. Thus, these stochastic firing delays, sampled from a negative exponential distribution, allow to automatically derived the stochastic process, i.e. a Continuous Time Markov Chain (CTMC), that describes the dynamics of the SSN model. Specifically, the CTMC states are isomorphic to SSN markings and the state changes correspond to the marking changes in the model.

Hereafter we recall the formal definition of SSN.

#### Definition 1.

(Stochastic Symmetric Net) An SSN is a nine-tuple:

$$ \mathcal{N}_{SSN}=\langle{P,T, \mathcal{C},I,O,\mathit{cd},\Theta,\omega,\mathbf{m}_{0}\rangle }   $$

where

- *P* and *T* are two disjoint finite non empty sets (representing places and transitions respectively).
- $\mathcal {C}=\{ C_{1}, \ldots, C_{n} \}$ is the finite set of basic color classes.
- $\mathit {cd}:\bigotimes _{i=1}^{n} \bigotimes _{j}^{e_{i}} C_{i}^{j}$ is a function defining the color domain of each place and transition (where $e_{i} \in \mathbb {N}$ is the number of occurrences of the class *C*_*i*_); for places it is expressed as Cartesian product of basic color classes, for transitions it is expressed as a list of variables with their types. Observe that a place may contain undistinguished tokens only or a transition may have no parameters, in this case their domain is *neutral*.
- *I*,*O*[*p*,*t*]:*cd*(*t*)→*Bag*[*cd*(*p*)] are the pre- and post- matrices, whose elements are in the form of the arc functions defined above.
- *Θ* is the vector of guards and maps each element of *T* into a standard predicate (*Θ*(*t*) may be the constant *true*, which is also a standard predicate).
- *ω*(*t*,*c*) is the function returning the rate of transition *t* assuming the firing of the instance 〈*t*,**c**〉.
- **m**_0_:*P*→*Bag*[*cd*(*p*)] is the initial marking, mapping each place *p* on a multiset on *cd*(*p*).

Assuming that all the transitions of the SSN are characterized by a Mass Action law, the intensity (also calles the transition speed) of 〈*t*,*c*〉 in marking *m* is defined as follows:

1 $$ \varphi(m,t,c)= \omega(t,c)  \prod_{\langle{p,c'}\rangle|\ p\in^{\bullet} \mathbf{t}\ \wedge\ c' \in \mathit{cd}(p)}  m[p][c']^{I[p,t](c)[c']}  $$

where *m*[*p*][*c*^′^] denotes the marking of place *p* for color *c*^′^.

In the literature, different techniques are proposed to solve (or analyse) the CTMC underlying an SSN; in particular, in case of very complex models, the so-called deterministic approach [15] can be efficiently exploited. According to this, in [16] we described how to derive a deterministic process, represented through a system of ODEs, which well approximates the stochastic behavior of an SSN model. In particular for each place *p* and possible color tuple *c*∈*cd*(*p*) we have the following ODE:

2 $$ \frac{dx_{p,c}(\nu)}{d\nu}=  \sum_{~~~~~~~~~\langle t',c'\rangle \in E(t',x(\nu))\ \wedge\ t' \in T} \varphi(x(\nu),t',c')(L[p,t'](c')[c])  $$

where *x*_*p*,*c*_(*ν*) is the average number of tokens of color *c* in the place *p* at time *ν*, *L*[*p*,*t*^′^](*c*^′^)[*c*]=*O*[*p*,*t*^′^](*c*^′^)[*c*]−*I*[*p*,*t*^′^](*c*^′^)[*c*], *T* is the set of transitions of the SSN, and *E*(*t*,*x*(*ν*)) the set of the enabled instances of *t* in *x*(*ν*), i.e. the vector of the average number of tokens at time *ν* for each place and possible color tuple. In this case Eq. 1 becomes

3 $$ \varphi(x(\nu),t,c)= \omega(t,c) \prod_{\langle{p_{j},c'}\rangle|\ p\in^{\bullet} \mathbf{t}\ \wedge\ c' \in \mathit{cd}(p_{j})} x_{p_{j},c'}(\nu)^{I[p_{j},t](c')[c]}.  $$

### Extended stochastic symmetric net

It is important to highlight that the reactions velocity can be defined by more complex laws than Mass Action (MA), for instance Michaelis Menten and Hill kinetics. In [17] the Extended Stochastic Petri Net (ESPN) formalism was presented to extend SPN with general functions which make easier to model reactions with more complex biological laws. Similarly, in this paper we propose a new formalism, called Extended Stochastic Symmetric Net (ESSN), which extends the SSN exploiting the same ideas discussed in the proposal of the ESPN formalism [17].

In details, the set of transitions *T* is split in two subsets *T*_*ma*_ and *T*_*g*_. The former subset contains all transitions which fire with a rate following a MA law. The latter includes instead all the transitions whose random firing times have rates that are defined as general real functions. Hence, we will refer to the transitions belonging to *T*_*ma*_ as standard transitions and as general transitions those in *T*_*g*_. For instance, considering the Fig. 1 again, the general transition is graphically represented as black box and is that simulating the myelin damage, i.e. *TeffKillODC*. In details, the function of the general transition is given in the Additional file 1.

#### Definition 2.

(Extended Stochastic Symmetric Net) An ESSN is a ten-tuple:

$$ \mathcal{N}_{ESSN}=\langle{ P,T,\mathcal{C},I,O,\mathit{cd},\Theta,\omega,\Omega,m_{0} }\rangle   $$

where

- $ P,\mathcal {C},I,O,\mathit {cd},\Theta,m_{0} $ are defined as in SSN (see definition 1).
- *T* is the set of transitions and is defined as *T*=*T*_*ma*_∪*T*_*g*_, with *T*_*ma*_∩*T*_*g*_=*∅*. Where $T_{ma}=\{t^{*}_{i}\}_{1 \leq i \leq n_{T_{ma}}}$ is the set of the $n_{T_{ma}}$ transitions whose speeds follow the MA law, and $T_{g}=\{\overline {t}_{i}\}_{1 \leq i \leq n_{T_{g}}}$ is the set of the $n_{T_{g}}$ transitions whose speeds are defined as general functions.
- *ω*(*t*,*c*) is the rate of transition *t*∈*T*_*ma*_ assuming the firing of the instance 〈*t*,*c*〉.
- *Ω*={*f*_〈*t*,*c*〉_}_*t*∈*T*∧*c*∈*cd*(*t*)_ is set grouping all the transition speeds ∀*t*∈*T*. In detail, with *t*∈*T*_*ma*_ then *f*_〈*t*,*c*〉_=*φ*(*cdot*,*t*,*c*), where *φ* is defined in Eq. 1.

Similarly to what discussed in Sec. *Petri Net and Stochastic Symmetric Net*, let $x_{p,c}(\nu) \in \mathbb {R}^{+}$ be the continuous approximation of the number of tokens in place *p* and color *c* so that the vector $x(\nu) \in \mathbb {R}^{n_{P}}\ $ is the marking of the ESPN at time *ν*.

Let define $ \hat {x} (\nu)= x(\nu)_{|^{\bullet } \mathbf {t}}\ $ as the subset of the marking *x*(*ν*) concerning only the input places to the transition *t*. Given 〈*t*,*c*〉 at the time *ν*, with transition *t*∈*T*=*T*_*ma*_∪*T*_*g*_, the firing of 〈*t*,*c*〉 will move tokens in state *x*_〈*p*,*c*〉_(*ν*) with speed $F(\hat {x}(\nu),t,c,\nu)$ defined as follows:

4 $$ {\begin{aligned} F(\hat{x}(\nu),t,c,\nu) := \left\{\!\begin{array}{cc} \varphi(\hat{x}(\nu),t,c), & t\in T_{ma},\\ f_{\langle{t,c}\rangle}(\hat{x}(\nu),\nu), & t\in T_{g}, \end{array}\right. \quad f_{\langle{t,c}\rangle}\in\Omega(t,c) \end{aligned}}  $$

where $\varphi (\hat {x}(\nu),t,c) $ is defined as in Eq. 3. Observe that $\varphi (\hat {x}(\nu),t,c)$ and $f_{\langle {t,c}\rangle }(\hat {x}(\nu),\nu)$ can depend only on the marking of the input places of transition *t* at time *ν*.

Finally the ODE characterizing the *p* and color tuple *c*∈*cd*(*p*) is defined as:

5 $$\begin{array}{*{20}l} \frac{dx_{p,c}(\nu)}{d\nu} &=  \sum_{\langle{\textbf{t}',\textbf{c}'}\rangle \in E(\textbf{t}',x(\nu))}  F(\hat{x}(\nu),\textbf{t}',\textbf{c}',\nu)(L[p,\textbf{t}'](\textbf{c}')[c])  \\ &=  \sum_{\substack{\langle{\textbf{t}',\textbf{c}'}\rangle \in E(\textbf{t}',x(\nu)) \\ \wedge \textbf{t}' \in T_{ma} }}  \varphi(\hat{x}(\nu),\textbf{t}',\textbf{c}')(L[p,\textbf{t}'](\textbf{c}')[c]) \\ & +  \sum_{\substack{\langle{\textbf{t}',\textbf{c}'}\rangle \in E(\textbf{t}',x(\nu)) \\ \wedge \textbf{t'} \in T_{g} }}  f_{\langle{\textbf{t}',\textbf{c}'}\rangle}(\hat{x}(\nu),\nu)(L[p,\textbf{t}'](\textbf{c}')[c]) \end{array} $$

where $\hat {x}(\nu)=x(\nu)_{|^{\bullet } \mathbf {t'}}$.

### Sensitivity analysis

Sensitivity analysis is broadly used in computational modelling to study which parameters affect mostly the variability of the outcomes produced by the model. Several approaches are proposed in the literature to achieve this task, such as Pearson correlation coefficient (CC) method (for linear relationships), Partial Rank Correlation Coefficient (PRCC) method (for non-linear and monotonic relationships) or Fourier Amplitude Sensitivity Test (FAST) method (for any non-linear relationships) [18, 19]. In this work we focus on a sampling-based method which combines Latin Hypercube Sampling (LHS) [20] with PRCC index. Practically LHS, a well-known stratified sampling method, is adopted to generate samples of the model input variables. Then the model is run N times in a chosen interval: one for each generated input variable sample combination. Finally PRCC between the generated input variables and the obtained model outputs are evaluated on the same chosen interval. In this way PRCC analysis and corresponding significance tests (i.e significant p-value) are used to identify key model parameters and to select time points which need an additional in-depth investigation. Specifically, PRCC values close to 1 (-1) identify positive (negative) monotone relationships between inputs and outputs; while the significance tests allow to discover those correlations that are important, despite having relatively small PRCC values.

## Model description

In this section we first describe how the healthy immune system achieves the immunosurveillance. Then we focus on the pathogenesis correlated to MS, highlighting the roles of the immune system cells, CNS cells, and EBV virus. Finally, we describe the structure of the model dividing it into seven modules.

### Healthy case

The immune system represents the entire compartment of cells leading the defense of the human body against potentially damaging foreign molecules and pathogens with a highly specific response to the encountered infectious agents. This specific response is conducted by a stringent selection and maturation of the naive T lymphocytes in the lymphoid organs (e.g. thymus), prior to exit in the periphery of the body as mature T lymphocytes cells. Consequently to the pathogens entrance, the naive T lymphocytes become activated T lymphocytes by Antigens Presenting Cells (APCs) via the T Cell Receptor (TCR). The activated T lymphocytes produce Interleukin-2 (IL2) an immunomodulating cytokine released for self-stimulating in order to duplicate and propagate their actions, via the binding of IL2 to the receptor IL2R, located on the surface of the cells. Thanks to this bond, activated T lymphocytes undergo the clonal expansion process (i.e. lymphocytes multiplication). During clonal expansion, a portion of the activated T lymphocytes are destined to become Effector T lymphocytes cells (Teff) and others as Memory T lymphocytes cells (Tmem). Thanks to the presence of Tmem cells, the responses to subsequent attacks from the same pathogen are faster and greater than the first one. Since the response of Teff is reversed when no longer needed, the Treg - response T lymphocytes - contributes to suppress the Teff cells activity. (see Additional file 1: Figure S1). In addiction, IL2 brings to the activation of another family of immune cells, named Natural Killer (NK) cells that act as host-rejection of infected cells and, in same cases, against auto-reactive Teff populations, T lymphocytes recognizing self-molecules as foreign [21].

### Multiple sclerosis

MS is an autoimmune disease in which the immune system removes the myelin sheath from neuronal axons of the CNS preventing the efficient transmission of the nervous signals. RRMS is the predominant type of MS in which the disease alternates phases of active neural inflammation and disease worsening (relapses) to remission phases in which there is a complete or partial lack of the symptoms. The occurrences of the relapses periods range from mild to severe, depending on the course and history of the disease. The occurrence and severity of relapses are the only measures to estimate the efficacy of a drug treatment.

Several studies agree with the idea that the damage at CNS is probably due to auto-reactive T lymphocytes which recognize the myelin as foreign. Indeed, during the relapse phase, the continuous attack of T lymphocytes leads to a progressive decrease of the quantity of myelin [22]. It is worth to note that in some cases the Oligodentrocytes cells (ODC) are able to partially recover the lost myelin if the damage is not excessive.

As already pointed out in the previous section, several T lymphocytes with different roles take part in the general immunosurveillance (e.g. Teff, Treg, Tmem). An imbalance in the T lymphocytes differentiation can lead to a strong or long-term response that goes beyond its original purpose. In fact, in literature it has been hypothesized that homeostasis of Treg and Teff cells may be crucial to prevent autoimmunity. Moreover, a breakdown of the peripheral tolerance mechanisms of Treg (represented, for example, by a lower duplication rate of Treg compared to Teff) can bring to the selection of self-reactive Teff cells leading to damage in the CNS [23]. The etiology of MS remains elusive. Some studies linked MS with signs of Epstein Barr Virus (EBV) infection [3, 24]. A hypothesis is that the EBV first infection as well as the reactivation of the latent infection can cause the activation of auto-reactive Teff lymphocytes through a process called molecular mimicry. Molecular mimicry would cause the activation of Teff lymphocytes that recognize an EBV virus protein. However, due to the structure similarity between the viral protein and myelin proteins, these Teff could also be able to attack myelin sheaths leading to a neural damage [3].

A very interesting case of study is the development and progression of MS in pregnant women. The RRMS pregnant women have a lower relapse rate until the delivery [25]. Indeed, during the pregnancy period the number of Treg cells increase to establish tolerance against fetal antigens. As a consequence, pregnancy represents a moment of immune tolerance and well-being for the patients.

### Relapsing-Remitting multiple sclerosis model

The cell and molecular interactions involved in the Relapsing-Remitting Multiple Sclerosis(RRMS) are described by the model showed in Fig. 2. Our model consists of 10 places and 22 transitions. For sake of clarity, the white transitions model mass-action functions, while the black transitions model different kinetics described in details in the Additional file 1. All the constants and numerical values associated with the transitions are reported in the Additional file 1: Tables S1 and S2. This model is organized in seven modules corresponding to the biological entities characterizing RRMS: Treg, Teff, EBV, NK, IL2, ODC, and DAC.

**1) Treg module.** The Treg cells are characterized by two places: the *Resting*_*Treg* and *Treg*. The transition *FromTimoREG* represents the arrival of new resting Treg cells from thymus. Its rate is defined in order to keep constant the number of resting cells. The transition *TregActivation* represents the activation of the resting Treg depending on the Teff cell number and EBV concentration, while *TregDeath* represents the death of Treg. The transition *TregKillsTeff* models the homeostatic regulation operated by Treg cells against self-reactive Teff cells, and *TregDup* models the Treg duplication.

**2) Teff module.** The second module is characterized by three places: *Resting*_*Teff*, *Teff*, and *EffectorMemory*. The transitions *FromTimoEFF*, *TeffActivation*, and *TeffDeath* behave similarly to those described in module 1, but they are referred to the Teff population.

The Teff proliferation takes place in two different manners called *Symmetrical* and *Asymmetrical* processes. These two possibilities are captured in the model by assuming that one happens with probability $p_{eff}^{dup}$ and the other with probability $p_{eff}^{mem}=1-p_{eff}^{dup}$. Given a replication speed named $r^{eff}_{dup}$, the transition *TeffDup_Sym* generates two Teff cells with the rate equals to $r^{eff}_{dup} * p_{eff}^{dup}$, for more details see Additional Material. Otherwise, the transition *TeffDup_Asym* takes place with a speed resulting from the product $r^{eff}_{dup} * p_{eff}^{mem}$ replicating one Teff cell into one T Memory effector cell and one Teff cell. The transitions *TeffKillsEBV* and *TeffKillsODC* encode the killing effect of Teff cells against EBV and ODC, respectively. Finally, *MemActivation* models the rapid activation of the Effector Memory depending on both the EBV and the Tmem concentrations.

The transitions *TeffKillsEBV* and *TeffKillsODC* encode the killing effect of Teff cells against EBV and ODC, respectively. Finally, *MemActivation* models the rapid activation of the Effector Memory depending on both the EBV and the Tmem concentrations.

**3) EBV module.** The third module describes the EBV behaviour. Transition *EBVinj* models the infection. The *TeffKillsEBV* transition summarizes all steps from antigen processing and presentation by EBV infected cells to Teff cells, to the activation of Teff cells.

**4) NK module.** In this modules the role of the NK cells is described. The transition *NKentry* models the arrival of new NK cells. The death of NK is then modeled by transition *NKDeath*. Transitions *NKKillsTeff* and *NKKillsTreg* encode the killing of self-reactive Teff and Treg cells respectively due to NK cells. Finally *NKdup* models the proliferation of the NK cells led by the presence of IL2.

**5) IL2 module.** The fifth module is focused on the *IL*2 role. *IL*2 is involved in the *Treg*, *Teff* and *NK* proliferation. All these types of cells consume IL2 which is produced by the transition *TeffActivation*.

**6) ODC module.** The sixth module encodes the ODC behaviour. The transition *TeffKillsODC* models the damage caused by Teff cells on ODC cells. When the myelination level reaches the lowest value, an irreversible damage occurs and the remyelinization is no more possible (i.e. the transition *Remyelinization* is permanently disabled by its guard).

To model this effect, we used the color class *Mye* encoding the myelination levels of ODC. *Mye* is divided into five static subclasses ranging from *Lmin* (no myelination) to *Lmax* (full myelination).

**7) DAC module.** In the last module the daclizumab behaviour is modeled through the place *DAC*. The drug administration is modeled by transition *DACinj*, while the pharmacokinetic inhibiting the expansion of Treg and Teff decreases the velocity of transitions *TregDup*,*TeffDup_Sym* and *TeffDup_Asym*. Finally, its degradation is modeled by the transition *DACDegradation*.

## Results

In this section we present the prototype computational framework developed to study the RRMS. Then, we describe how our model was calibrated to mimic the real behaviours in healthy and MS subjects. Finally we discuss also the results coming from two case studies in which we investigated the effect of the daclizumab administration on MS patients and the modification of the MS evolution due to the occurring of a pregnant condition.

### Framework architecture

**Fig. 3:**
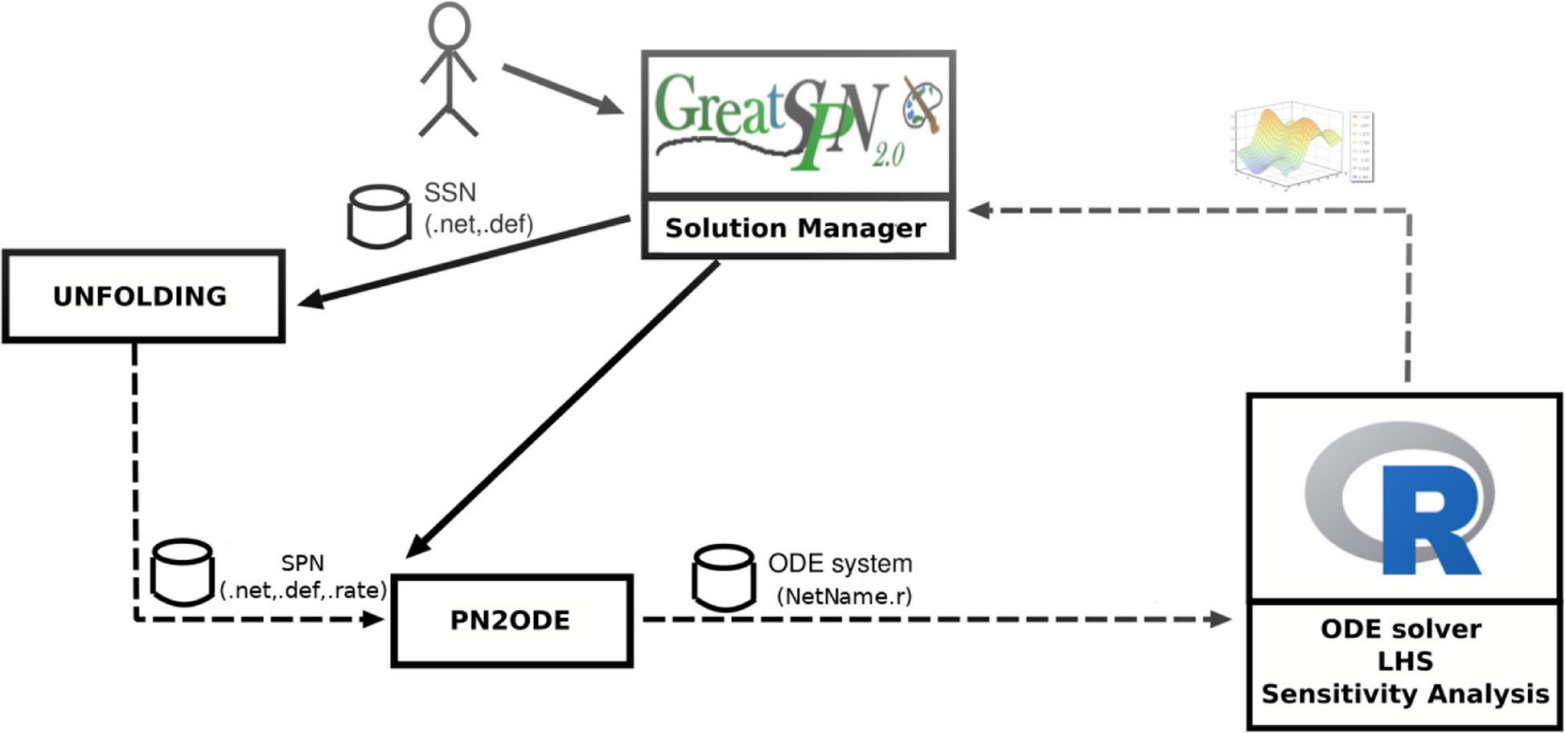
Framework structure. Outline of the prototype framework combining GreatSPN suite with R. The components are shown by rectangles, component invocations by solid arrows, models/data exchanges by dashed arrows

The prototype framework specifically developed for this project consists of a few modules which interact with GreatSPN [26], a software suite for modelling and analyzing complex systems using the PN formalism and its extensions. The architecture of this framework is depicted in Fig. 3. The GreatSPN GUI is used to construct the ESSN model. First the coloured portion of a model “NetName” (similar to that depicted in Fig. 2) is drawn on the GUI interface, then the “Unfolding” module is called to transform the coloured model in a basic Petri Net representation where all the tokens are assumed to be indistinguishable and the files “NetName.net” and “NetName.def” are produced. Moreover the generic firing rate functions characteristic of the ESSN formalism must be specified in a separate file called “name.rate”. Then the files “NetName.net” and “NetName.def” and “NetName.rate” are used by the module “PN2ODE” to automatically derive the corresponding system of ODEs producing a file “NetName.r” with a format suitable for being processed by the “R” software. Finally the “R” environment is used for the solution of the set of ODEs, for carrying out the sensitivity analysis with respect to model parameters, and for computing interesting results in terms of numerical values and diagrams. As the whole framework is not yet completely integrated with GreatSPN, calling the “Unfolding” and “PN2ODE” modules, as well as the interaction with the “R” software, must be done using command-line directives. GreatSPN extended with this prototype framework can be downloaded at https://github.com/greatspn. Instead, all the R files and the GreatSPN files of the net are freely available at https://github.com/qBioTurin/ESSNandRRMS/tree/master/DeterministicModel.

### Model calibration for healthy and mS individuals

The model calibration was addressed to select the input values (transition parameters, and the concentrations of EBV and DAC) leading outputs of the model towards the values obtained from observing the behaviour of the specific quantities both in healthy and MS affected subjects. The calibration is performed using the LHS with PRCC index to identify which parameters have more impact on the model outcomes. Then, the identified parameters were thoroughly investigated in the healthy and MS individuals.

From our model (without the *DAC* module, Fig. 2) a system of 13 ODEs with nine input parameters is derived. The values of these nine parameters were sampled by means of LHS method. Hence, 5000 parameter combinations were generated using a *uniform* distribution whose ranges are showed in the second column of the Additional file 1: Table S1.

For all the simulations, we assumed as initial marking the following parameters consistent with a space of 1*mm*^3^ of blood and 4*mm*^3^ of neural tissue: 500 ODC with level *L*_*max*_ of neuronal myelinization, 1687 resting Teff cells, 63 resting Treg cells, 375 NK cells and 1000 IL2 molecules, and zero cells in the other places (see Additional file 1: Table S3).

Moreover, we defined the disease occurrence when the *L*_*min*_ level of neuronal myelinization is reached for each ODC cell, representing an irreversible damage. Then, five virus injections are simulated at regular times (every two months), introducing into the system 1000 EBV copies per injection. Finally, model solutions were calculated for each parameter combination over one year interval, [0,365] days.

Analyzing the 5000 trajectories generated, three scenarios have been identified: (i) the occurrence of the MS, represented by a huge number of dead ODC cells; (ii) the complete remission of the MS disease, characterized by a low number of dead ODC cells and with a complete elimination of the EBV virus; (iii) the partial remission of the MS disease specified by a partial elimination of the EBV virus. The Additional file 1: Figure S2 reports the EBV and ODC dynamics generated considering different set of parameters.

**Fig. 4:**
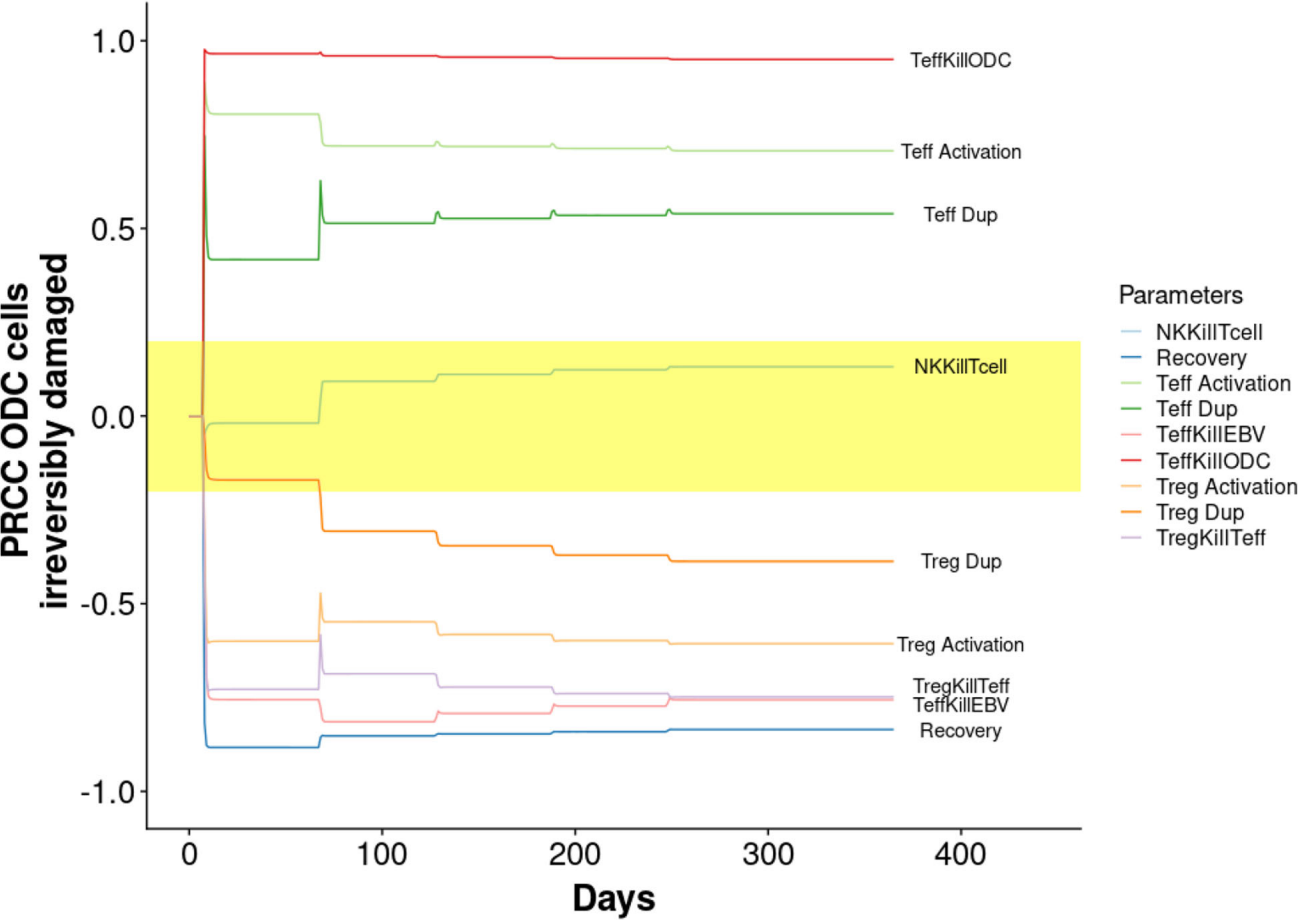
Sensitivity analysis. PRCCs over the whole time interval for each model parameter is reported. Yellow area represents the zone of non-significant PRCC values

On these trajectories the PRCC analysis was applied to identify key model parameters affecting the system behaviour. The PRCCs values are calculated for each parameter over the entire time period. Figure 4 shows the PRCC values for all the model parameters over the time interval considered.

**Fig. 5:**
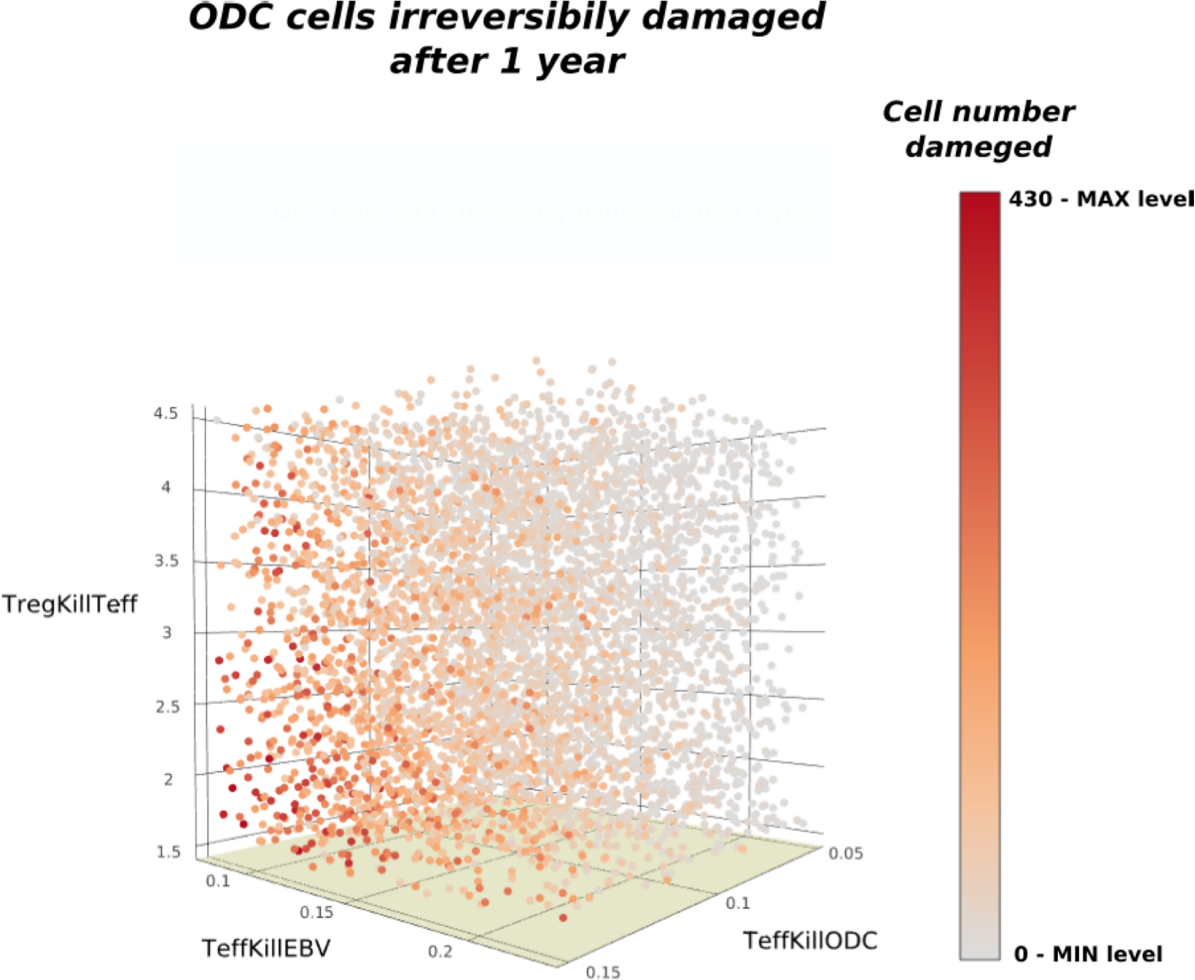
Parameters scatter plot. 3D scatter plot of the ODC irreversible damaged at the fixed time 365 versus the*TeffKillODC*, *TregKillTeff*, *TeffKillEBV* parameters variation

The rates associated with transitions *TeffKillODC*, *TregKillTeff*, *TeffKillEBV* and *Recovery* result to be the crucial parameters affecting the *ODC* behaviour. Figure 5 reports a scatter plot in which each point corresponds to a generated trajectory, its color represents the percentage of irreversible ODC damaged at the final time point (i.e. a grey color corresponds to a lowest percentage of damaged ODC and a red color to highest one). The simulations are performed changing the rates of *TeffKillEBV* (in the x-axis), the *TregfKillTeff* (in the y-axis) and *TeffKillODC* (in the z-axis). A few number of irreversible damaged ODC are obtained increasing the *TregfKillTeff* rate and the decreasing the others two rates.

**Fig. 6:**
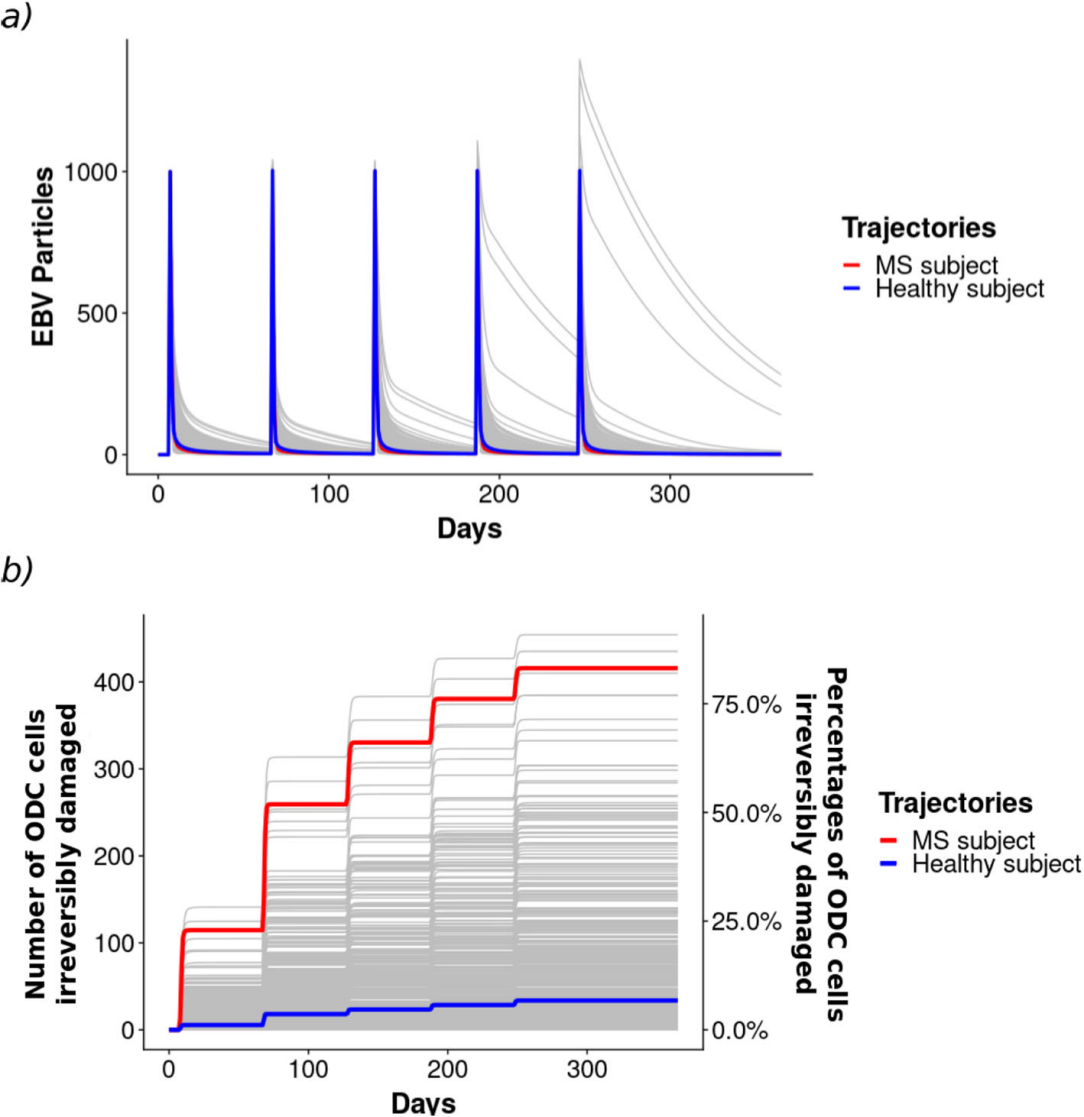
Parameters choice. A set of the 500 trajectories generated by LHS of the EBV virus (**a**) and the ODC cells with an irreversible damage (**b**) over the whole time interval varying the *TeffKillODC*, *TregKillTeff*, and *TeffKillEBV* transition parameters

The key parameters identified were deeply studied exploiting the LHS method computing 500 new combinations varying only *TeffKillEBV*, *TregfKillTeff* and *TeffKillODC*. We defined two sets of parameters (see Additional file 1: Table S4), one for the MS patients and one for the healthy subjects, see Fig. 6.

The MS patients are modelled by a set of parameters which maximizes the ODC damage maintaining the Treg and Teff cell numbers consistent with those measured in the reality, see red trajectories in Fig. 6a and b panels.

**Fig. 7:**
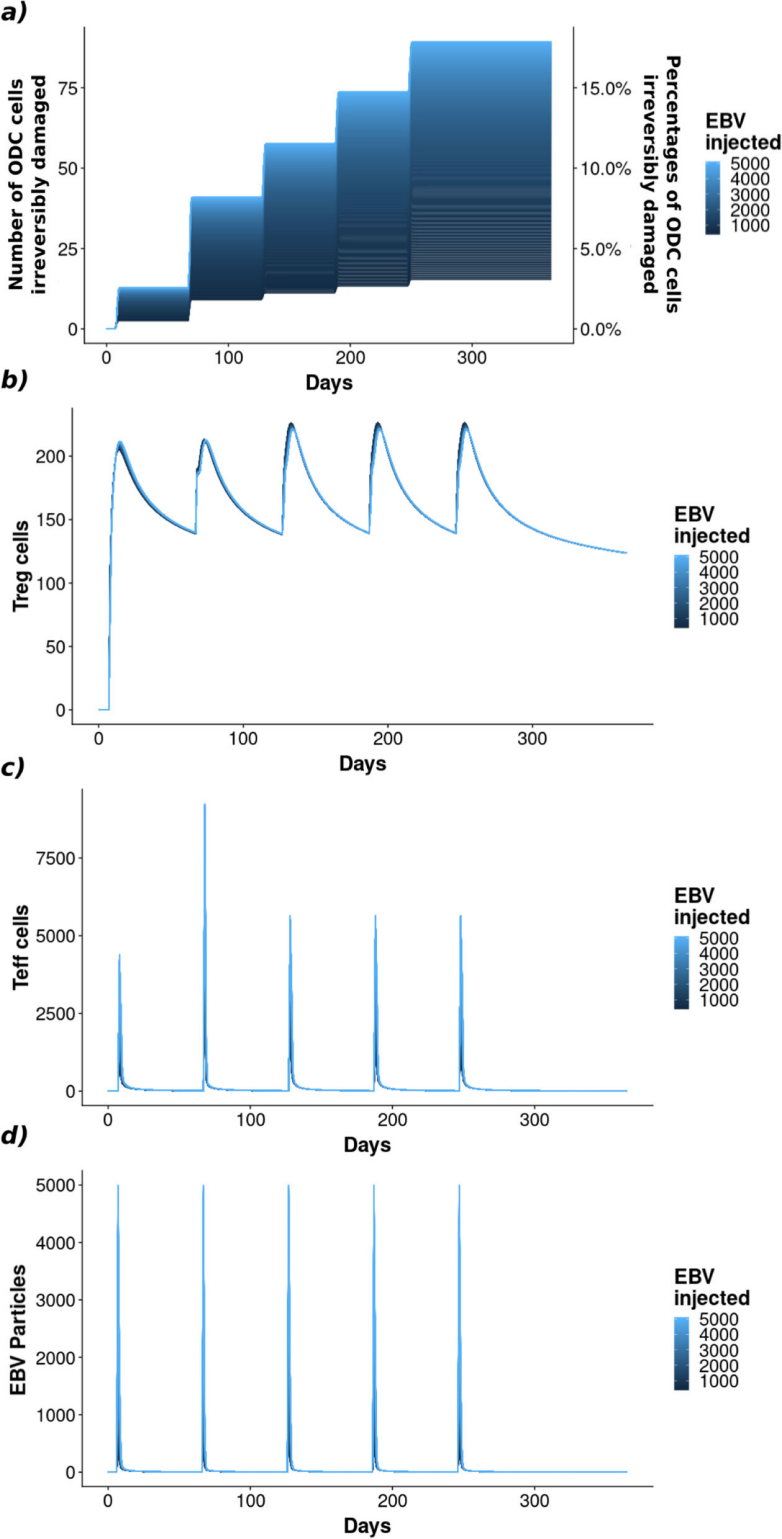
Effect of EBV quantities. Different injections of EBV (**d**) are considered to check if the Teff-Treg (**c**-**b**) regulatory loop is able to control the virus spreading minimizing the irreversible damages to the ODC cells (**a**)

For the healthy cases, we selected the trajectory providing a Teff-Treg regulatory balance able to control the spread of the EBV virus and to minimize the irreversible damage to ODC cells, even if the amount of EBV in each injection is substantially increased, see blue trajectories in Fig. 6, panels a and b. In particular, we performed 500 simulations varying the amount of EBV injected in a range of [1000−5000 *particles*/*mm*^3^]. From Fig. 7 it is possible to observe that, even for large quantities of EBV injected, the percentage of irreversibly damaged ODCs reaches 17% (Fig. 7a). This value is very small if compared with respect to the 77% of irreversibly damaged ODCs in the case of the disease occurrence. Moreover, independently of the quantity of EBV injected, Teff are able to eliminate EBV completely (Fig. 7d), and the abundance of EBV does not drastically affect the number of effectors or regulators in the system (Fig. 7b,c).

### DAC therapy

To investigate the effect of the DAC therapy in our RRMS model calibrated for MS patients, we simulated the DAC administration at the 53rd day after the first EBV injection. Our results are reported considering two important aspects in the modulation of a therapy: the drug dose and the drug degradation time. The quantity of *DAC* administrated per injection and the *DAC* cells deterioration were studied by means of LHS method. The values of these two parameters are then sampled according to two uniform distributions whose ranges are reported into the Additional file 1: Table S1.

**Fig. 8:**
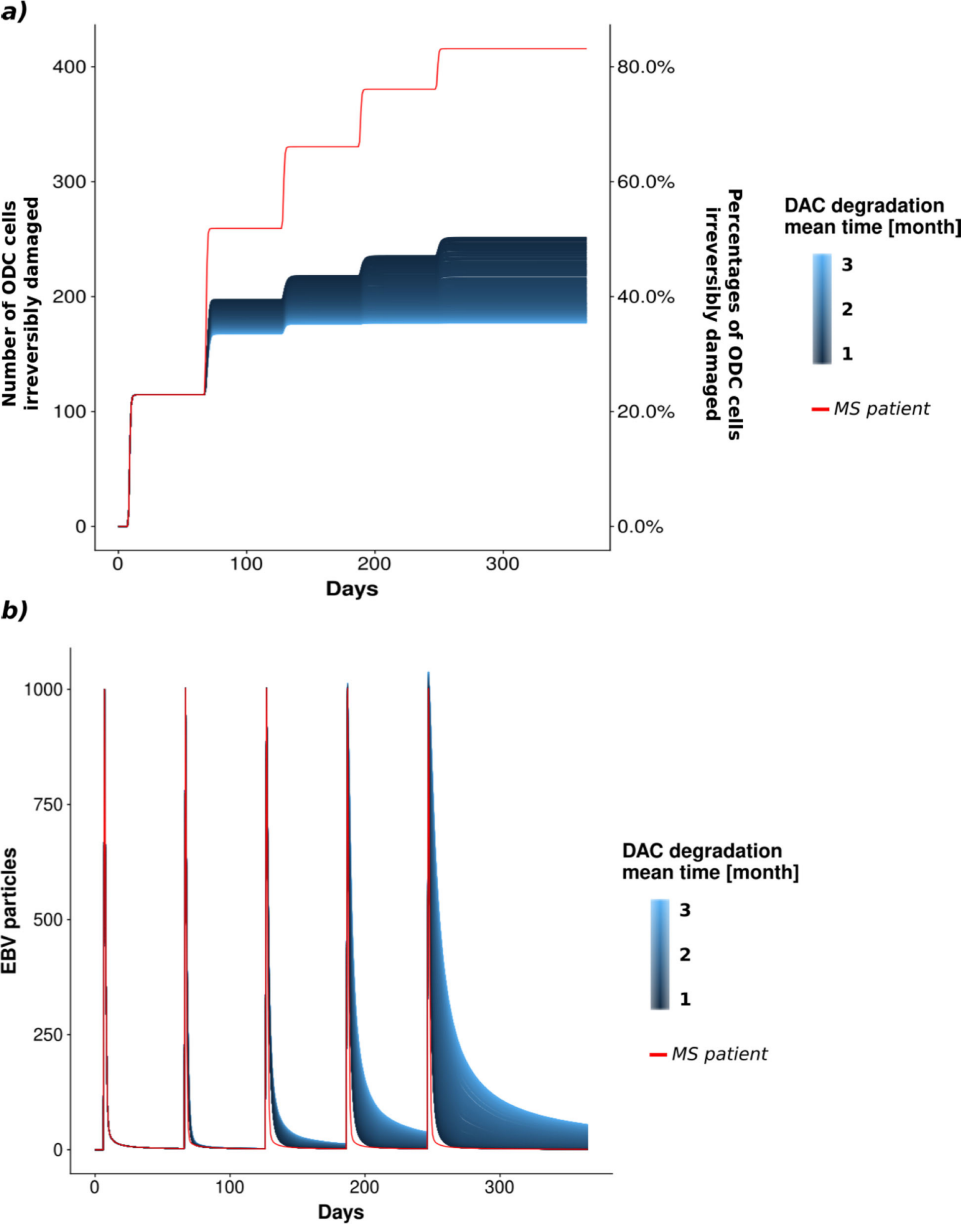
Varying the DAC degradation rate. *ODC* and *EBV* trajectories colored depending on *DAC* degradation rate (expressed in months). The red line represents the starting sample without drug administration

From the LHS analysis we clearly observed that the drug degradation time has a greater impact on the elimination of EBV virus than the amount of DAC administered, see the number of ODC irreversibly damaged in Additional file 1: Figure S3. Therefore, we decided to focus our attention on the *DACDegradation* parameter variation. Knowing that the half-life of DAC was detected around 22 days, we considered that a complete degradation of DAC ranges between 30 and 90 days [27]. The results of the simulations are reported in Fig. 8a in which it is possible to appreciate that a greater DAC permanence has the effect of reducing the number of irreversibly damaged ODC cells with respect to the case in which no therapy was considered (red line). Moreover, it is interesting to note that the RRMS model with DAC injections highlights a decrease of the long term ability of the immune system to eliminate EBV Fig. 8b. Finally, in Additional file 1: Figure S4 is reported the trend of the NK cells that increase with respect to the DAC degradation rate.

### Pregnancy

In this subsection we investigate the RRMS in pregnant women. As already pointed out before, pregnancy was associated with fewer relapses in RRMS and reduced activity of disease in autoimmune encephalomyelitis (EAE). Beneficial effects of pregnancy are thought to be related to pregnancy-associated changes in the maternal immune system. One of the observations is that Treg cells increase in number establishing the fetal tolerance and conferring a temporary protection to women with RRMS [28, 29].

According to the literature, we modelled the pregnancy condition changing the proportion between the activate Treg cells and the activate Teff cells decreasing the Teff activation rate and increasing the Treg activation rate proportionally to the pregnancy phase [29]. Three pregnancy phases, corresponding to the three trimesters, have been simulated. When a new trimester begins, we increased the ratio of *TregActivation* rate to *TeffActivation* rate; while at delivery time both rates return to their initial values.

**Fig. 9:**
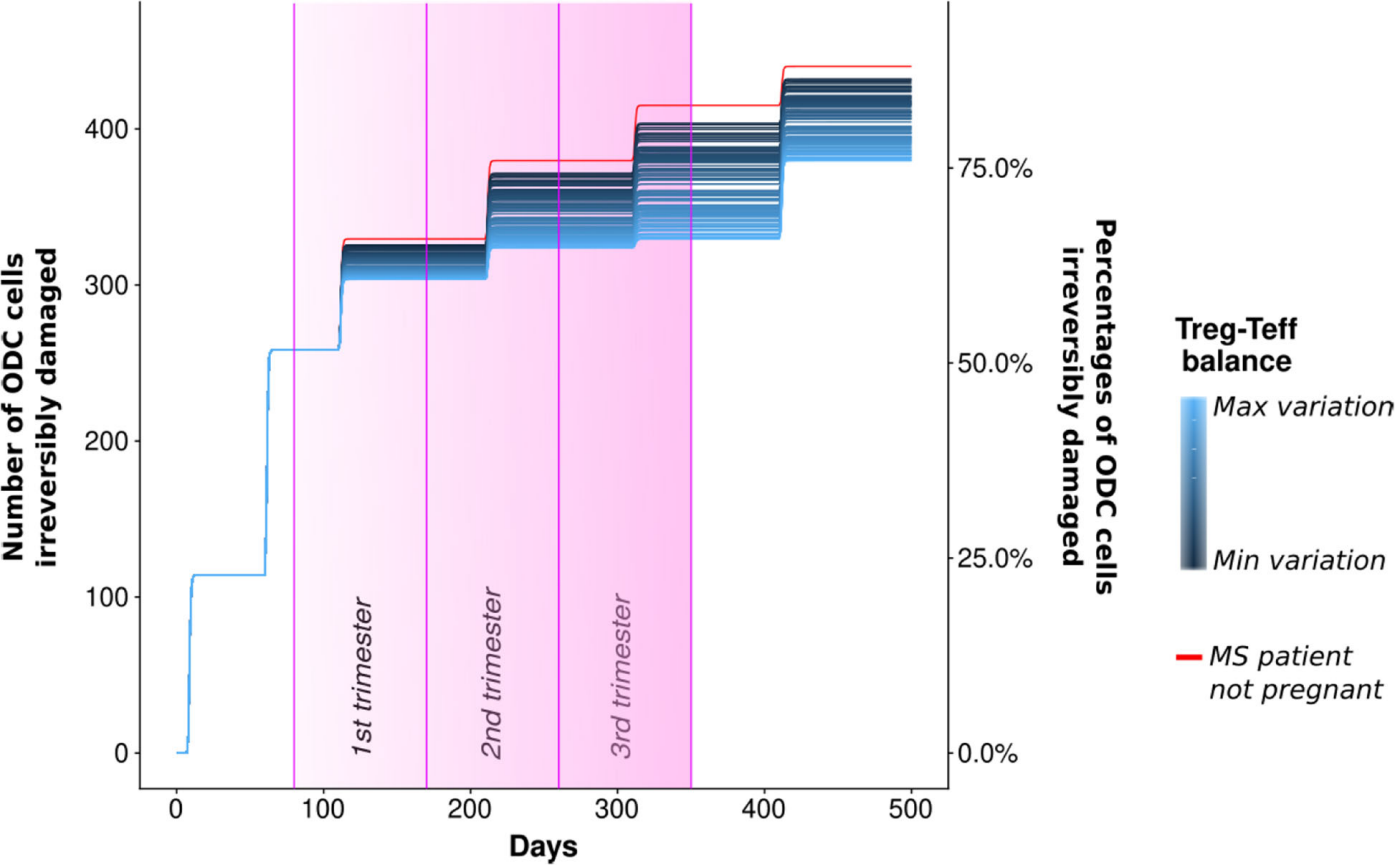
Pregnant woman case: ODC. The ODCs irreversibly damaged considering the pregnant woman case of study. 100 trajectories colored depending on different variations of the *TregActivation* and *TeffActivation* parameters. The red line represents the starting sample without pregnancy. Furthermore each trimester another variation is applied to these parameters in order to represent the increasing of the maternal immune system

Thus we simulated 100 different scenarios with a increasing variation of parameters, obtaining different levels of protection from ODC damage. As expected, the model behaviour shows a substantial reduction of the ODCs damage (see Fig. 9).

Regarding the immune system cells, we observed that Treg cells increase during pregnancy and then suffer a sharp decline at the time of delivery. The same effect, but in the opposite direction is showed on Teff cells. It is interesting to note that a rebound of Teff is reported in the week following pregnancy, see Additional file 1: Figures S5.

## Discussion

Because of nowadays the computational modelling is widely recognized to succeed in helping scientists in the study of the complex mechanisms of different diseases, in this paper we presented a new computational methodology and an associated model to better elucidate the dynamics behind the RRMS. Indeed, RRMS represents a very challenging case study, due to the complexity of the disease which involves many different biological agents, ranging from molecular to environmental factors.

We exploited the descriptive power of Extended Stochastic Symmetric Nets to provide a graphical representation of the complex biological system in a compact and parametric way. Moreover, we used LHS method with PRCC index to calibrate the model parameters. Hence, we showed the ability of the model to reproduce the typical oscillatory behavior relating to the onset of RRMS by supposing a breakdown of the cross-balance regulation mechanisms at the peripheral level. Moreover, the simulation of DAC injections in the RRMS model can help scientists to define the mechanisms of actions of this drug and to theorize the possible causes of its observed sideeffect on the patients. Instead, the experiments simulating RRMS in pregnant women can contribute to define the mechanisms at the basis of the variation of the Treg and Teff cells.

A challenging issue in the definition of the RRMS model is the calibration of the transition parameters and EBV and DAC concentrations. The LHS with PRCC index identified *TeffKillODC*, *TregKillTeff*, *TeffKillEBV* as the most critical parameters to the model outcomes. This result agrees with our expectation since these parameters play a central role in the disease progression. In the analysis of the EBV behaviour in a healthy subject, it is interesting to note the effect of immune memory which increases the number of activated Teff cells from the time of second injection(see Additional file 1: Figure S6). In particular, thanks to the faster activation of the Tmem cells with respect to the Teff cells, from the second EBV injection it is possible to observe a more rapid virus annihilation. Indeed, Tmem cells have a faster activation (i.e. since they already have the memory of a previous contact with the EBV) than Teff cells, leading to a more rapid virus annihilation during the relapses.

In the first set of our experiments we inspected the effect of the DAC therapy in our model calibrated for reproducing the behaviors of RRMS patients. A greater amount of latent EBV is present in the system with respect to the case in which no therapy was considered.

In the DAC therapy module there are two parameters form whom depend the elimination of EBV virus: the DAC degradation time and the DAC concentration. Using the parameter sensitivity analysis, we identified the drug degradation time as the crucial parameter, while the DAC concentration has ho effect on the EBV treatment. Indeed, this slight effect of the DAC concentration in the treatment of MS patients was recently described by Gold and co-workers [30]. In this paper the authors described a clinical trial involving 76 centers in which the MS patients have been treated by subcutaneous injections of DAC HYP 150 mg or 300 mg, or placebo, every 4 weeks for 52 weeks. The annualised relapse rate was lower for patients given DAC HYP (150 mg or 300 mg) than for those that received the placebo, However, no significance difference in terms of relapse between the different DAC doses was reported. Finally, the DAC analysis revealed the secondary effects of the DAC immunosuppressive therapy that can actually increase susceptibility to secondary infections.

The second set of experiments were devoted to study the effect of RRMS in pregnant patients. The mechanisms at the basis of a partial MS remission during the pregnancy are not fully understood yet, leading this case particularly interesting. During pregnancy, the maternal immunotolerance to the fetus induced Treg proliferation and reduced the relapse rate, therefore our model predicts a reduction of ODC damage. Our results are in line with what comes to us from biological knowledge and clinical observations since the resetting of the immune system is what significantly influences the course of the disease during pregnancy and also has been related with clinical manifestation of increased relapses associated with the post-partum period. From our results it is possible to appreciate the difference between the ODC cells irreversibly damages in the case of MS no pregnant patients and MS pregnant patients (specially in the case of max variation of the Treg-Teff balance). This difference increases from the first trimester to the time of delivery then returns to become not significant.

At the best of our knowledge, this is the first paper in which the colored Petri Nets and the sensitivity analysis are systematically used to study RRMS.

## Conclusions

In this paper we provide a promising application of a computational framework based on Colured Petri Nets and sensitivity analysis to perform *in silico* experiments helping to improve the understanding of the RRMS disease, possibly giving some indications that may ameliorate the clinical management. The results of the simulations give us the opportunity to identify the key parameters involving in the modulation of the effect of the DAC therapy. Moreover, the simulation in the pregnancy in MS patients mimics the resetting of the immune system. As future works we will expand the model including different cell populations of the immune system for a better integration between adaptive and innate immunity. Moreover, we plan to insert new color classes in the model to encode the spatial coordinates of all entities in a cubic tissue portion. In the context of precision medicine, we may exploit the model to predict the patient-specific outcome when the DAC therapy is administered. This will require an initial model calibration with patient-specific clinical data. Indeed, our model contains a set of crucial input parameters regarding the concentration of immune cells in different modules (e.g. *Teff module*, *NK module*, and *ODC module*) that can be retrieved from clinical data of each patient. Then, these values can be used to personalize the model in order to obtain the DAC administration schedule tuned with respect to the patient characteristics.

## Supplementary information

**Additional file 1** Supplementary File

## Data Availability

All data generated and analyzed during this study are included in this published article and its supplementary information files. Moreover, all the R files and the GreatSPN file of the net are freely available at https://github.com/qBioTurin/ESSNandRRMS.
