# Supplementary material for: A computational approach based on the colored Petri net formalism for studying multiple sclerosis
Source: BMC Bioinformatics. 2019 Dec 10;20(Suppl 6):623. doi: 10.1186/s12859-019-3196-4 (PMC6904991; doi:10.1186/s12859-019-3196-4)
Supplement: Supplementary file 1 — Additional file 1 Supplementary File [file 12859_2019_3196_MOESM1_ESM.pdf]

1 Supplementary File

In this section we describe in details the firing rate function associated with the generic transitions in our RRMS model. These transitions model the following biological events: i) the killing of a cell, e.g., *TregKillsTeff* or *TeffKillsODC*, ii) the entry of cells into the system, such as *EBVinj*, iii) the activation of T cells, e.g., *TeffActivation*, and iv) the duplication of a cell,e.g., such as *TeffDup*.

Let us recall the following notations: $f_{\langle t,c\rangle}(\hat{x}(\nu),\nu)$ is the speed of the transition $t \in T_g$ and $\hat{x}(\nu)$ represents the vector of the average number of tokens for all the input places of t. For brevity when the function will not depend on the color instance c then we will omit it reporting just the transition t, i.e. $f_t(\hat{x}(\nu),\nu)$. All the general transitions of the model are now explained in details and all the constants are summarized in Tables 1 and 2.

- *EBVinj*, *DACinj* inject into the system specific quantities of EBV and DAC respectively at fixed time points;
- *FromTimoREG*, *FromTimoEFF*, and *NKentry* are the transitions which keep in a constant range the number of *RestingTreg*, *RestingTeff*, and NK respectively. They are defined as

$$f_{FromTimoREG}(x_{ResTreg},\nu) = q_{RestTreg} * (1 - x_{ResTreg}/63);$$
$$f_{FromTimoEFF}(x_{ResTeff},\nu) = q_{RestTeff} * (1 - x_{ResTeff}/1687);$$
$$f_{NKentry}(x_{NK},\nu) = q_{NK} * (1 - x_{NK}/375),$$

  where $x_{ResTreg}$, $x_{ResTeff}$, and x_{NK} are the numbers of cells in the input places (i.e. *RestingTreg* for *FromTimoREG*, etc) at time ν. Then $q_.$ represents the quantity injected in the output place to preserve the cell quantity, i.e. 63 for the *RestingTreg*, 1687 for the $RestingTeff$ and 375 for the NK (see table 3).
- *TregActivation* and *TeffActivation* transitions model the activation of the Teff and Tref cells. In particular, these transitions are defined of type general to simulate a reduced Teff activation velocity when the virus presence decreases, and a Treg activation velocity which is proportional to the number of Teffs and inversely proportional to the number of EBV particles (allowing the Teff to annihilate the virus). So the functions are defined as

$$f_{TregActivation}(\hat{x}(\nu),\nu) = r_{TregA} * \frac{x_{Teff}}{(x_{Teff} + x_{EBV} + 1)} * x_{ResTreg};$$
$$f_{TeffActivation}(\hat{x}(\nu),\nu) = r_{TeffA} * (1 - exp(-\frac{x_{EBV}}{C_{EBV}})) * x_{ResTeff},$$

  where r_{TregA} and r_{TeffA} are the activation constant rates for the Treg and Teff respectively. In case of the $TregActivation$ transition, the vector $\hat{x}(\nu)$ of its input places $RestingTeff, EBV$ and $Teff$ consists of the variables $x_{ResTreg}, x_{EBV}, x_{Teff}$ respectively. Differently the $\hat{x}(\nu)$ of $TeffActivation$ transition is characterized by $x_{ResTeff}$ and x_{EBV}. Finally the constant C_{EBV} is related to the EBV particles and it is defined to reduce the activation rate with the decreasing of the virus presence.

- *MemActivation* is defined as

$$f_{MemActivation}(\hat{x}(\nu), \nu) = \begin{cases} 0 & \nu < t_{2inj}, \\ r_{MemA} * x_{Mem}(\nu) & \nu \geq t_{2inj}, \end{cases}$$

where

$$r_{MemA} = 2 * r_{TeffA} * (1 - \exp(-\frac{x_{Mem}(\nu)}{C_{Mem}}) * (1 - \exp(-\frac{x_{EBV}}{C_{EBV}}),$$

and t_{2inj} is the time corresponding to the second EBV injection. We are considering the velocity of this transition as zero $\forall \nu < t_{2inj}$, since the T Memory effectors start to react after the first virus occurrence. $\hat{x}(\nu) = (x_{Mem}(\nu), x_{EBV}(\nu))$ is the marking vector storing the number of T Memory effectors and EBV particles respectively at time ν. C_{Mem} and C_{EBV} constants are related to the Memory and EBV cells needed to slow down the activation rate with the decreasing of EBV and Memory cells. This is due to the necessity of leaving a minimum number of T Memory effectors into the system. So when in the system there are a large number of EBV particles and of T Memory effectors, the activation speed reaches its maximum given by twice the velocity of the Teff cells, r_{TeffA}.

- All the transitions modeling the killing of a specific cell are defined as follows:

$$\forall t \in \{ \textit{TregKillsTeff}, \textit{TeffKillsODC}, \textit{TeffKillsEBV}, \textit{NKKillsTcell} \}$$

then

$$f_t(\hat{x}(\nu), \nu) = \frac{1}{x_{tot}} * r_t * \prod_i \hat{x}_i(\nu),$$

where $\prod_i \hat{x}_i(\nu)$ is the product of the average numbers of tokens in the input places of the transition t, r_t is the constant rate related to the transition t, x_{tot} is the total number of cells at time ν, and $\frac{1}{x_{tot}}$ represents the probability that a specific meeting between two different cells is occurred.

- *TregDup* transition models the Treg duplication depending proportionally on the amount of IL2 and inversely proportionate on the number of DAC cells (to simulate the reduced duplication velocity during the daclizumab therapy), and it is defined as:

$$f_{TregDup}(\hat{x}(\nu), \nu) = \eta_{TrD}(\hat{x}(\nu), \nu) * x_{Treg} * x_{IL2} * \frac{1}{x_{tot}},$$

with

$$\eta_{TrD}(\hat{x}(\nu), \nu) = r_{TregDup} * (1 - exp(-\frac{x_{IL2}}{C_{IL2}})) * (exp(-\frac{x_{DAC}}{C_{DAC}})),$$

where $r_{TregDup}$ is the constant Treg duplication rate, $\hat{x}(\nu) = \{x_{Treg},\ x_{IL2},\ x_{DAC}\}$ and C_{IL2} and C_{DAC} are the constant related to the IL2 and DAC cells to

slow down the duplication velocity with an increasing number of DACs and a decreasing number of IL2 proteins.

- Considering the Teff duplication event we have to distinguish two possible cases: 1) the Teff symmetric duplication with probability p_{eff}^{dup} and a Teff asymmetric duplication, implying the T Memory effector differentiation, with probability $p_{eff}^{mem} = 1 - p_{eff}^{dup}$. This is modeled exploiting two different transitions: *TeffDup_Sym* and *TeffDup_Asym*. So let us define

$$r_{dup}^{eff} = \eta_{TeD}(\hat{x}(\nu), \nu) * x_{Teff} * x_{IL2} * \frac{1}{x_{tot}}$$

then these two transitions are defined as:

$$f_{TeffDup\_Sym}(\hat{x}(\nu), \nu) = p_{eff}^{dup} * r_{dup}^{eff}$$

and

$$f_{TeffDup\_Asym}(\hat{x}(\nu), \nu) = p_{eff}^{mem} * r_{dup}^{eff},$$

with

$$\eta_{TeD}(\hat{x}(\nu), \nu) = r_{TeffDup} * (1 - exp(-\frac{x_{IL2}}{C_{IL2}})) * (exp(-\frac{x_{DAC}}{C_{DAC}})).$$

Where $r_{TeffDup}$ is the constant Teff duplication rate, $\hat{x}(\nu) = \{x_{Teff},\ x_{IL2},\ x_{DAC}\}$.

2 Figures

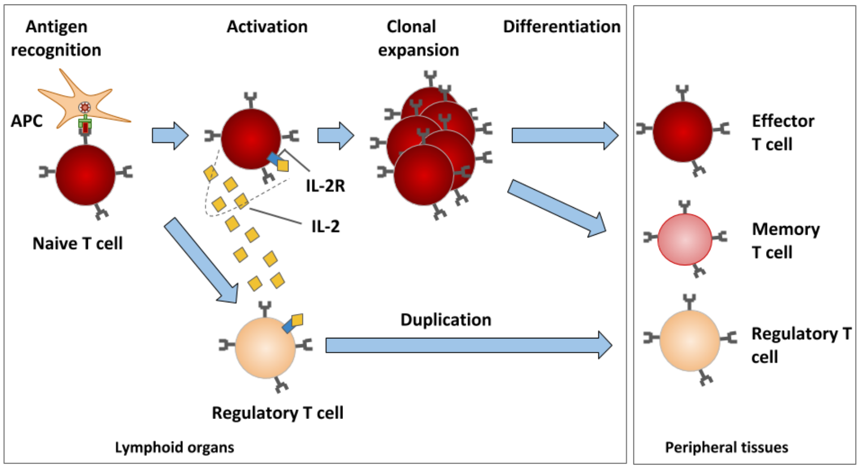

Figure 1: **T Lymphocytes activation.** The naive T Lymphocytes are activated through the bound of the TCR receptor with an Antigen Presenting Cell (APC). Then, T Lymphocytes start to produce and release IL2 in the environment. IL2 is simultaneously internalized by T Lymphocytes in order to self-stimulate the duplication and differentiation in Effector T cells (Teff), Memory T cells (Tmem) and Regulatory T cells (Treg).

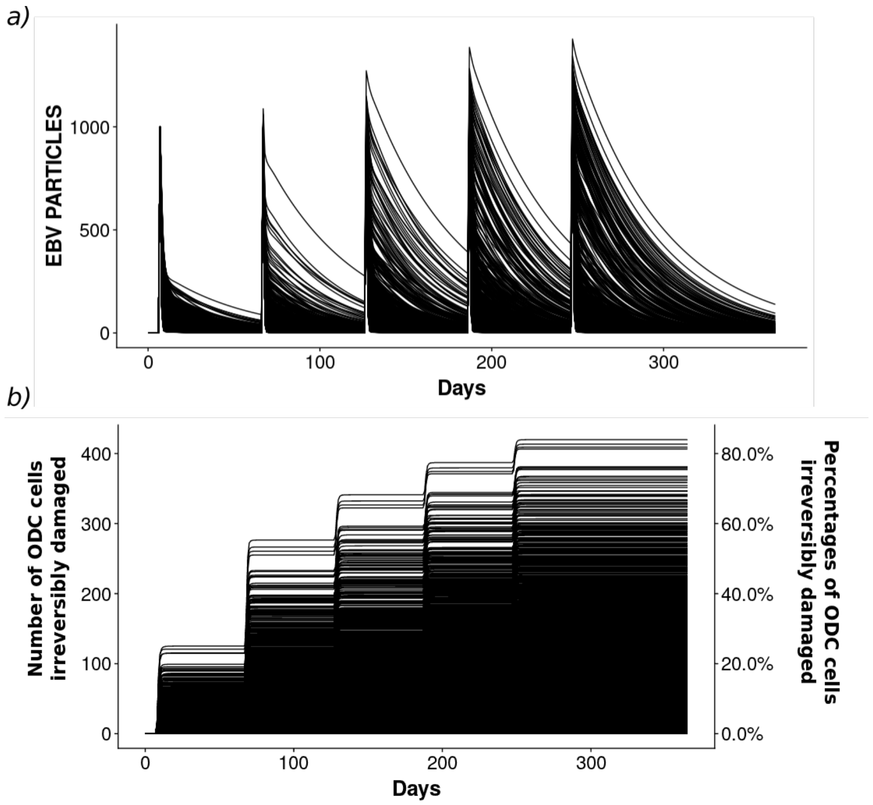

Figure 2: **EBV and ODC dynamics.** A subset of the 5000 trajectories generated by LHS of the EBV cells (a) and the ODC cells with an irreversible damage (b) over the whole time interval

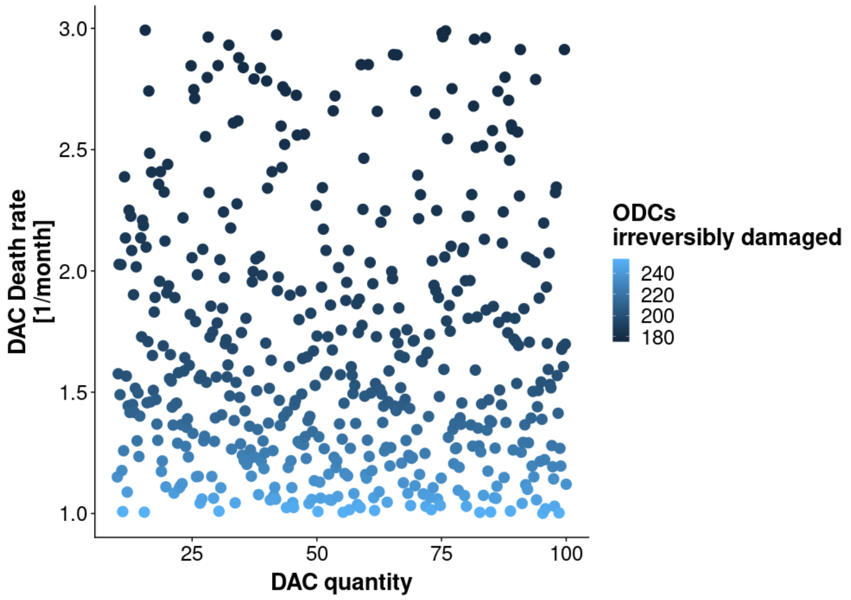

Figure 3: **Varying the quantity of DAC injected and its degradation.** Scatter plot of the ODC irreversibly damaged variable at the fixed time 365 depending on the DAC injected (x-axis) and the DAC death rates (y-axis). The colour depends on the number of ODCs irreversibly damaged. The number of ODC is strongly dependent by the DAC degradation: the decrease of the number of damaged ODC is more influenced by an increase of the permanence time of DAC drug in the body.

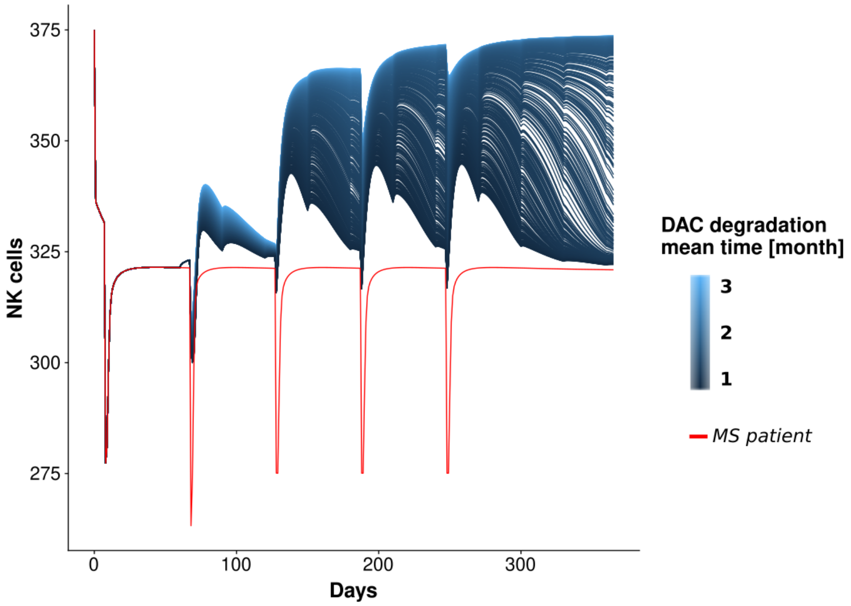

Figure 4: **Result of the variation of DAC degradation rate in NK cells.** NK trajectory colored depending on DAC degradation rate (expressed in months). The red line represents the starting sample without drug administration.

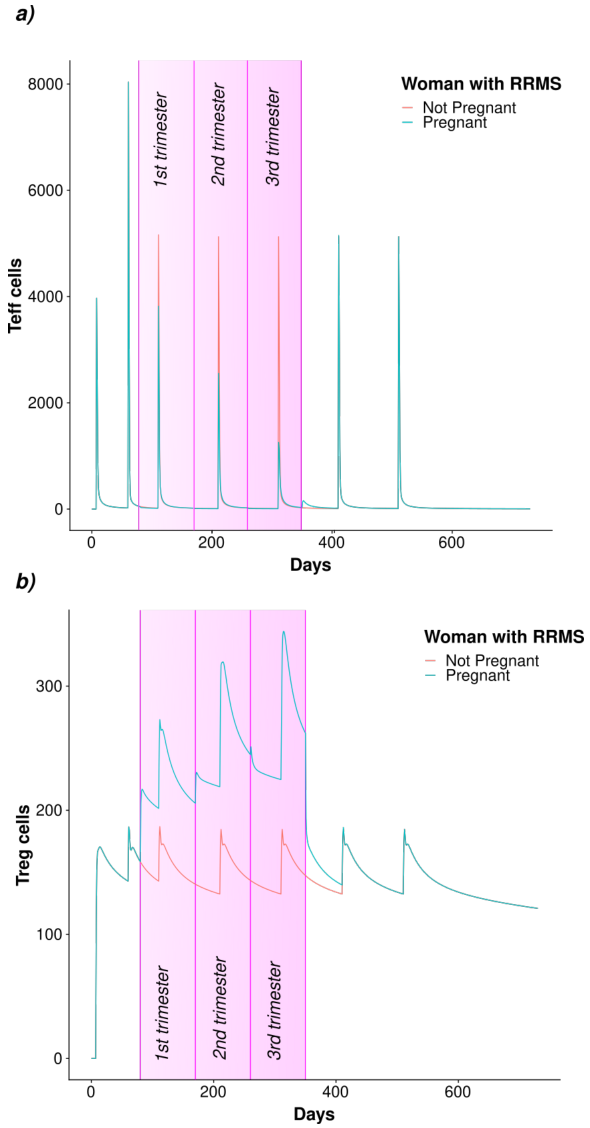

Figure 5: **Pregnant woman case: Teff and Treg.** The Teff a) and Treg b) dynamics before, during and after the pregnancy. The red line represents the starting sample without pregnancy.

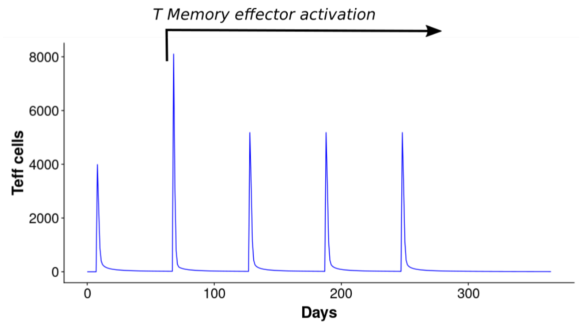

Figure 6: **Teffs considering an MS subject.** The Teff dynamics considering an MS subject.

3 Tables

Table 1: List of the model fixed and unknown parameters with their corresponding values or (in the latter case) ranges on whose the Uniform distribution is defined.

Transitions/events	Parameters	Range
Treg Death	r_{TregD}	$1/24\ h^{-1}$
Teff Death	r_{TeffD}	$1/24\ h^{-1}$
NK Death	r_{NKD}	$1/24\ h^{-1}$
NK Dup	r_{NKDup}	$1/24\ h^{-1}$
Teff Activation	r_{TeffA}	$[0.2, 0.6]\ h^{-1}$
Treg Activation	r_{TregA}	$[0.1, 0.3]\ h^{-1}$
Treg Dup	$r_{TregDup}$	$[0.045, 0.135]\ h^{-1}$
Teff Dup	$r_{TeffDup}$	$[0.25, 0.75]\ h^{-1}$
TeffKillODC	r_{TeKodc}	$[0.05, 0.15]\ h^{-1}$
TregKillTeff	r_{TrKTe}	$[1.5, 4.5]\ h^{-1}$
TeffKillEBV	r_{TeKebv}	$[0.075, 0.225]\ h^{-1}$
Recovery	r_{rec}	$[0.075, 0.225]\ h^{-1}$
NKKillTcell	r_{NKkTc}	$[0.05, 0.15]\ h^{-1}$
DACDeath	r_{DacD}	$[0.0004, 0.001]\ h^{-1}$
DACinjection	r_{DacInj}	$[5, 100]\ h^{-1}$

Table 2: List of the model constants.

Constant	Value
$q_{RestTreg}$	20
$q_{RestTeff}$	500
q_{NK}	100
C_{EBV}	1000
C_{Mem}	200
C_{DAC}	$(DACinjected)/log(.1)$ [1]
C_{IL2}	200
C_{Tcell}	200
C_{Teff}	200
p_{eff}^{dup}	2/3
p_{eff}^{mem}	1/3

[1]DACinjected represents the quantity of DAC injected per time and with this formula we estimate automatically the constant in order to have $exp(-DACinjected/C_{DAC}) = .1$, i.e. the T-cells duplication rate is reduced of the 90% when all the DAC particles are present.

Table 3: List of the cell numbers used in the model.

Cell	Value	Reference
$TLymphocytes$	$[3 * 10^3 cells/mm^3]$	[1, 2]
$RestingTeff$	$[1687cells/mm^3]$	[3–6]
$RestingTreg$	$[63cells/mm^3]$	[7]
NK	$[375cells/mm^3]$	[3–6]
ODC	$[125cells/mm^3]$	[8]
$EBVinfection$	$[50 - 70days]$	[9]

Table 4: Parameters used for simulating the Healthy version (first row) and Sick version (second row) of the disease.

**Transition**	Teff Activation	Treg Activation	Treg Dup	Teff Dup	TeffKillODC	TregKillTeff	TeffKillEBV	Recovery	NKKillTcell
Healthy	0.4	0.2	0.09	0.5	0.1	3	0.15	0.1	0.1
Sick	0.4	0.2	0.09	0.5	0.15	1	0.1	0.1	0.1

4 References

Author details

References

1. Al-Mawali, A., Pinto, A.D., Al-Busaidi, R., Al-Lawati, R.H., Morsi, M.: Comprehensive haematological indices reference intervals for a healthy omani population: First comprehensive study in gulf cooperation council (gcc) and middle eastern countries based on age, gender and abo blood group comparison. PloS one **13**(4), 0194497 (2018)
2. Warny, M., Helby, J., Nordestgaard, B.G., Birgens, H., Bojesen, S.E.: Lymphopenia and risk of infection and infection-related death in 98,344 individuals from a prospective danish population-based study. PLoS medicine **15**(11), 1002685 (2018)
3. Santagostino, A., Garbaccio, G., Pistorio, A., Bolis, V., Camisasca, G., Pagliaro, P., Girotto, M.: An italian national multicenter study for the definition of reference ranges for normal values of peripheral blood lymphocyte subsets in healthy adults. Haematologica **84**(6) (1999)
4. Bisset, L.R., Lung, T.L., Kaelin, M., Ludwig, E., Dubs, R.W.: Reference values for peripheral blood lymphocyte phenotypes applicable to the healthy adult population in switzerland. European journal of haematology **72**(3), 203–212 (2004)
5. Choi, J., Lee, S.J., Lee, Y.A., Maeng, H.G., Lee, J.K., Kang, Y.W.: Reference values for peripheral blood lymphocyte subsets in a healthy korean population. Immune network **14**(6), 289–295 (2014)
6. Saathoff, E., Schneider, P., Kleinfeldt, V., Geis, S., Haule, D., Maboko, L., Samky, E., Souza, M.d., Robb, M., Hoelscher, M.: Laboratory reference values for healthy adults from southern tanzania. Tropical Medicine & International Health **13**(5), 612–625 (2008)
7. Somerset, D.A., Zheng, Y., Kilby, M.D., Sansom, D.M., Drayson, M.T.: Normal human pregnancy is associated with an elevation in the immune suppressive cd25+ cd4+ regulatory t-cell subset. Immunology **112**(1) (2004)
8. Segal, D., Schmitz, C., Hof, P.R.: Spatial distribution and density of oligodendrocytes in the cingulum bundle are unaltered in schizophrenia. Acta neuropathologica **117**(4), 385 (2009)
9. Balfour Jr, H.H., Holman, C.J., Hokanson, K.M., Lelonek, M.M., Giesbrecht, J.E., White, D.R., Schmeling, D.O., Webb, C.-H., Cavert, W., Wang, D.H., et al.: A prospective clinical study of epstein-barr virus and host interactions during acute infectious mononucleosis. Journal of Infectious Diseases **192**(9) (2005)
